# Encapsulation of miRNA and siRNA into Nanomaterials for Cancer Therapeutics

**DOI:** 10.3390/pharmaceutics14081620

**Published:** 2022-08-03

**Authors:** Mina Zare, Rakesh Pemmada, Maya Madhavan, Aswathy Shailaja, Seeram Ramakrishna, Sumodan Padikkala Kandiyil, James M. Donahue, Vinoy Thomas

**Affiliations:** 1 Center for Nanotechnology and Sustainability, Department of Mechanical Engineering, National University of Singapore, Singapore 117581, Singapore; mina.zare@helsinki.fi (M.Z.); seeram@nus.edu.sg (S.R.); 2 Department of Food and Nutrition, University of Helsinki, 00014 Helsinki, Finland; 3 Departments of Materials Science and Engineering, Biomedical Engineering, University of Alabama at Birmingham (UAB), Birmingham, AL 35294, USA; rpemmada@uab.edu; 4 Department of Biochemistry, Government College for Women, Thiruvananthapuram 695014, India; 5 Department of Pediatrics, Duke University School of Medicine, Durham, NC 27710, USA; aswathy.shailaja@duke.edu; 6 Post Graduate Department of Zoology, Government College, Madappally 673102, India; sumodanpk@gmail.com; 7 School of Medicine, University of Alabama at Birmingham, Birmingham, AL 35294, USA; jdonahue@uabmc.edu; 8 Center for Nanoscale Materials and Biointegration (CNMB), Center for Clinical and Translational Science (CCTS), University of Alabama at Birmingham (UAB), Birmingham, AL 35294, USA

**Keywords:** RNA delivery, nanocarriers, cancer therapy, nanomedicine

## Abstract

Globally, cancer is amongst the most deadly diseases due to the low efficiency of the conventional and obsolete chemotherapeutic methodologies and their many downsides. The poor aqueous solubility of most anticancer medications and their low biocompatibility make them ineligible candidates for the design of delivery systems. A significant drawback associated with chemotherapy is that there are no advanced solutions to multidrug resistance, which poses a major obstacle in cancer management. Since RNA interference (RNAi) can repress the expression of genes, it is viewed as a novel tool for advanced drug delivery. this is being explored as a promising drug targeting strategy for the treatment of multiple diseases, including cancer. However, there are many obstructions that hinder the clinical uses of siRNA drugs due to their low permeation into cells, off-target impacts, and possible unwanted immune responses under physiological circumstances. Thus, in this article, we review the design measures for siRNA conveyance frameworks and potential siRNA and miRNA drug delivery systems for malignant growth treatment, including the use of liposomes, dendrimers, and micelle-based nanovectors and functional polymer–drug delivery systems. This article sums up the advancements and challenges in the use of nanocarriers for siRNA delivery and remarkably centers around the most critical modification strategies for nanocarriers to build multifunctional siRNA and miRNA delivery vectors. In short, we hope this review will throw light on the dark areas of RNA interference, which will further open novel research arenas in the development of RNAi drugs for cancer.

## 1. Introduction

Cancer is a group of diseases that cause abnormal and uncontrolled cell division with the characteristics of invasion and metastasis [1]. In spite of great advances in present-day clinical sciences, malignant growth remains difficult to treat and has become the main cause of death around the world (around 13% of all deaths) [2]. Chemotherapeutic agents have explicit limitations with respect to treating cancer, the most important one being side effects and the development of multidrug resistance against them. Essentially, the non-particulate nature of most of these restorative agents brings about huge harm to normal cells [3]. These agents additionally require widespread dissemination in the body, resulting in reduced infiltration into the tumor, causing toxicity to normal tissues and further necessitating the need for the recurrence of dosing [4]. Numerous hazardous effects related to the non-selective cytotoxicity of chemotherapeutic agents are unavoidable as chemotherapy is fundamentally a systemic treatment [5].

To facilitate an innovative provision to overcome the issues of existing chemotherapeutic agents, nanotechnology is being tapped for cancer therapy due to its enormous potential. For the purposes of higher drug loading, intracellular take-up, and to focus extensively on the growth of tissues, an assortment of nanocarrier stages with sizes of 10–200 nm is favored [6,7]. A huge assortment of hybrid nanomaterials of inorganic and organic origins is currently being formed with biomolecular species incorporating multi-stimuli-responsive supramolecular congregations for the delivery of the designed nanomaterials [7]. Considering the enormous effect that nanotechnology has had on the healthcare sector, another field named “nanomedicine” dedicated to this specific sector has evolved [8]. Nanomedicine has effectively had a huge effect on delivering drugs more productively, diagnosing diseases more quickly and sensitively, and conveying antibodies through aerosols and nanoparticles [9]. Aside from these, the mediation of nanotechnology has fundamentally decreased the time needed from testing to the treatment.

The delivery of miRNA to the target cells faces a huge challenge as these particles must travel long distances through nuclease-rich blood compartments [10]. Therefore, miRNA degradation in the extreme biological environment of the blood circulation must be prevented with high-strength miRNA carriers. Even though mRNA and siRNA have commendable potential as therapeutic agents, research on their simultaneous delivery is limited to an extent. Ball et al. co-formulated siRNA and mRNA in a single lipidoid nanoparticle (LNP) formulation and optimized the treatment of diseases related to distorted gene upregulation and downregulation [11].

RNA interference is a very efficient tool for gene silencing where noncoding RNA sequences are used to guide the degradation of target mRNA. Noncoding RNAs, which were thought to be non-functional in earlier times, were later discovered to regulate transcription and translation, thereby making them therapeutically significant in many diseases including cancer. Of particular importance is sncRNA (small noncoding RNA), which comprises microRNA (miRNA), small interfering RNA (siRNA), piwi-interacting RNA (piRNA), small nucleolar RNA (snoRNA), and transfer RNA (tRNA).

RNAi is a mechanism that is mainly involved in the manipulation of gene expression, naturally operating in all eukaryotes and is also evolutionarily conserved [11]. miRNA and siRNA are central to RNAi therapeutics, which are widely gaining momentum at present. siRNA-mediated gene silencing is initiated when dsRNA produced by pathogenic organisms or the transcription of endogenous, or even exogenous, genes enters the cell when it is processed by Dicer to generate siRNA [12]. Central to this mechanism is the action of the core enzyme Dicer, an endoribonuclease that recognizes the exogenous double-stranded RNA through its helicase domain. The newly formed siRNA, when it binds to the RNA-induced silencing complex (RISC), the Argonaute 2 (Ago2) component of RISC, degrades the sense strand of the siRNA. The remaining guide strand undergoes degradation when it binds to its complementary mRNA, resulting in gene silencing.

**Figure 1:**
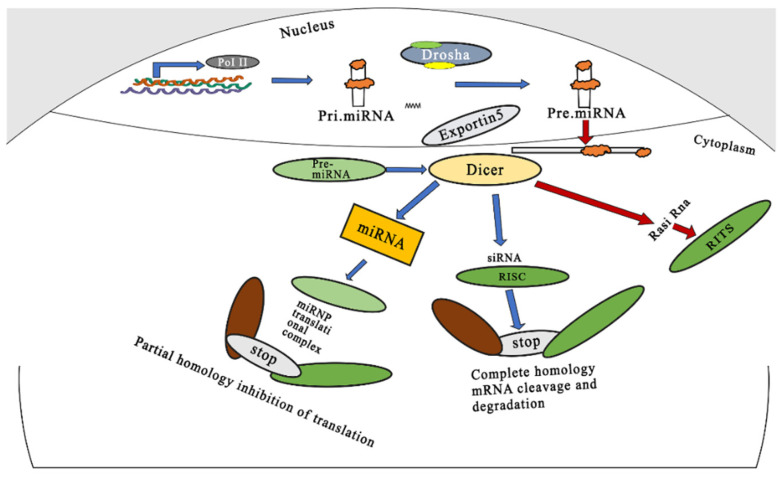
Schematic representation of mechanism of gene silencing by miRNAs and siRNAs. Adapted and modified from [12] under the Creative Commons license.

On the other hand, miRNA-mediated gene silencing is initiated by the generation of pri-miRNA (primary miRNA) by the transcription of miRNA genes catalyzed by polymerase II (Pol II). The pri-miRNA is converted to pre-miRNA (precursor miRNA) by the action of Drosha [13]. The pre-miRNA that enters the cell with the help of Exportin5 is processed to miRNA by Dicer. The double-stranded miRNA then binds to RISC, during which the sense strand of the miRNA is released and discarded. The guide strand binds to the target mRNA, but only with partial complementarity, and hence multiple targets can be silenced by this approach. Figure 1 illustrates the generalized mechanism of siRNA- and miRNA-mediated gene silencing.

Figure 1 represents the RNA interference mechanism. siRNA-mediated gene slicing is initiated with dsDNA production, followed by the transcription of endogenous and exogenous genes, the formation of siRNA by the action of Dicer, the binding of the newly formed siRNA to RISC, and the degradation of the strand of siRNA by the Ago2 component RISC, finally resulting in the degradation of complementary mRNA. For miRNA-mediated gene silencing, the mechanism is initiated by the formation of pri-miRNA by polymerase II, then pri-mRNA is converted to pre-miRNA by Drosha. Double-stranded miRNA then binds to RISC followed by the degradation of the target mRNA.

miRNA therapeutic strategies can be broadly divided into two types: those that involve miRNA suppression and those that involve miRNA replacement. In miRNA suppression, the target gene expression is enhanced by the inhibition or sequestering of miRNAs. Different approaches to miRNA suppression such as the use of inhibitors [14], masks, and sponges are available to achieve this. On the contrary, the use of miRNA mimics, agomirs, pre-miRNA, or miRNA-expressing plasmids can lead to the upregulation of endogenous miRNA activity, thereby resulting in gene silencing.

Around 60–90% of protein-coding genes are regulated by miRNAs, which are archived in miRbase [15]. Each mature miRNA has a characteristic sequence, called the canonical form. However, isomiRs, isomeric forms of miRNA with variations in their sequence, have been identified by small-RNA deep sequencing, which may be generated due to alternative processing of RNase III family members of classes II and III (Drosha/Dicer) [16], post-transcriptional RNA editing [17], and non-templated nucleotide addition [18].

In the present article, we discuss the latest developments in siRNA and miRNA drug delivery systems for malignant growth treatment, including the use of liposomes, dendrimers, and micelle-based nanovectors, as well as functional polymer–drug delivery systems. We believe that new nanoparticles and a combination of existing nanoparticle-based strategies can overcome the remaining challenges. This improvement can help in formulating miRNA- and siRNA-based therapy in the future, especially for developing personalized medicine for patients’ diseases based on their miRNA expression profiles. Additionally, this review sums up the advancements in and challenges to nanocarriers for siRNA delivery and remarkably centers around the most critical modification strategies for nanocarriers to build multifunctional siRNA and miRNA delivery vectors.

## 2. Challenges Associated with RNAi Therapeutics

**Figure 2:**
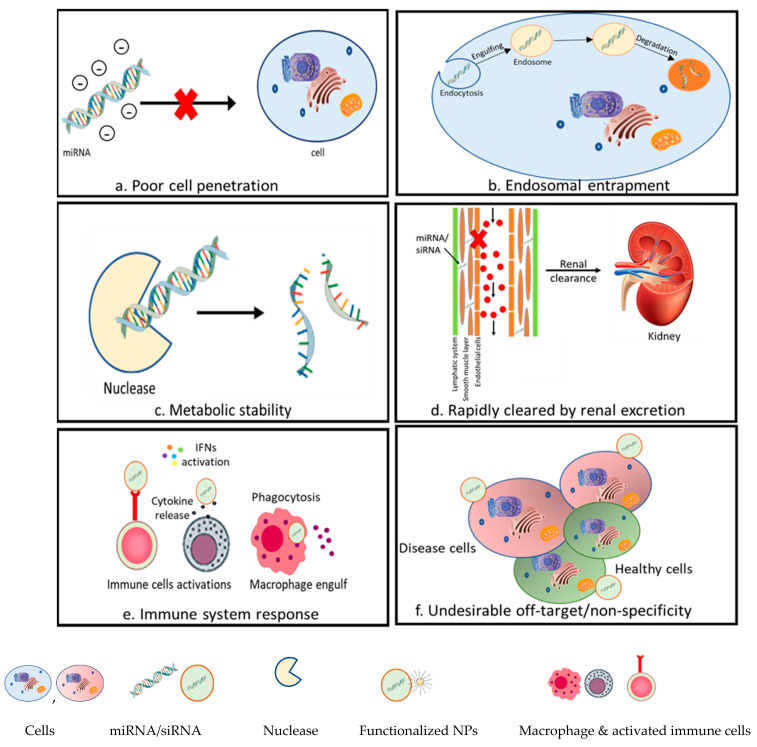
The challenges of in vivo miRNA/SiRNA delivery.

siRNA-based treatments offer incredible potential for use in therapy for numerous neglected clinical requirements, such as the management of viral infections, neurological diseases hereditary disorders, and cancer. However, there are a few obstructions that limit the full capability of RNAi-based methodologies relating to siRNA instability, undesirable off-target effects, and nonspecific activation of the innate immune system. Additionally, there are obstacles regarding siRNA delivery into cells, such as clearance via the reticuloendothelial system (RES); hindrances presented by endothelial cells; and failure to accomplish endosomal escape, thus preventing the siRNA from reaching the site of cytosolic activity. A number of these challenges are rectified with the introduction of nanocarrier drugs, especially liposome- and micelle-based ones. Figure 2 illustrates the challenges of miRNA/SiRNA delivery in vivo. In recent years, numerous studies have been conducted that point to the important role of miRNAs in the repression of genes in different diseases and thence prospects of RNAi in the development of novel clinical applications and therapeutic strategies. However, several challenges still need to be addressed.

The requirement for protected and powerful delivery techniques remains a critical challenge to better understanding the expansive capability of siRNA-based therapeutics. Usually, unmodified siRNAs are often immunogenic, and they are reluctant to cross the cell membranes as they are temperamental in the circulation system [13]. Therefore, in order to carry siRNA to its site of activity without adverse impacts, chemical modification of potential delivery materials are needed. To address the difficulties of in vivo conveyance, an expansive variety of materials is under investigation, including polymers [14], lipids [15], peptides [16], antibodies [17], aptamers [18], and small molecules [19].

### 2.1. Poor Cell Membrane Penetration

The use of viral and non-viral vectors are the two major strategies used for therapeutically delivering siRNA into the target cells. The initial concerns regarding siRNA-mediated gene silencing mainly concerned the dsRNAs, which, when delivered exogenously, may activate the interferon pathway leading to non-specific miRNA degradation and apoptosis. To avoid this undesirable effect, attempts were made to directly deliver siRNA into the cells. The lipid bilayer is the fundamental barrier that prevents invading RNA from entering the cells. Moreover, RNA oligomers, being polyanionic in nature, face severe challenges in crossing the cell membrane [20].

Lipid-based nanoparticle formulations are most commonly used for the delivery of siRNA [21] because they offer many advantages such as ease of preparation, versatility, improved bioavailability, and low toxicity, etc. The circulation time and efficiency of these nanoparticles can be sufficiently increased by PEGylation [22].

Even if the miRNAs are delivered by the vectors, the intracellular trafficking of miRNAs is generally initiated in the early endosome compartment. Then, the early endosomes combine with late endosomes followed by the transfer of their content, and, eventually, the endosomal content moves to the lysosomes. The acidification and activity of various nucleases in the lysosomes leads to the degradation of siRNA/miRNA. To avoid this degradation, miRNA must escape from the endosomes and translocate to the cytoplasm. This endosomal escape is one of the challenges in miRNA therapeutics. Electrostatic interactions between the cationic lipid component used in lipoplexes and lipid nanoparticles (LNPs) and the phospholipid bilayer of the endosomal membrane are reported to mediate endosomal escape [23] via the ‘proton sponge’ effect [24]. Recently, Lee et al. have reported a novel DNA-inspired nanomaterial platform for siRNA delivery that can enhance endosomal escape [25].

### 2.2. Off-Target Effects

The off-target effects generated by siRNA were initially reported by Jackson et al. [19]. The similarity between the guide strand of siRNA and miRNA can lead to miRNA-like off-target effects, associated with siRNA-mediated RNAi [26]. Nucleobase modifications in the guide strand of siRNA have been found to produce a significant reduction in miRNA-like off-target potency [27]. In another report, glycol nucleic acid (GNA) modifications in the seed region of the siRNA sense strand have been reported to limit the off-target effects [28].

High-dose single miRNA treatments can cause unwanted off-target effects, mainly due to the random binding properties of miRNAs. This can lead to potential toxicities and reduce the effects of the therapeutics. This problem can be overcome by using a strategy of mixing low-dose miRNAs that synergistically regulate the expression of the same target gene [29]. For example, the miRNAs miR-34a and miR-15a/16 act together to induce cell cycle arrest in non-small cell lung cancer (NSCLC) cells. Administering a combination of both miRNAs that regulate similar pathways and share common targets may potentially increase their therapeutic impact [30].

### 2.3. Metabolic Stability and Bioavailability

Although the specificity, design simplicity, and potency of small oligonucleotide molecules make them suitable for use in targeting many diseases such as cancer, viral infections, and autoimmune disorders, the small size and negative charge of these molecules present unfavorable pharmacokinetics. The susceptibility of naked oligonucleotides to degradation by endogenous nucleases [31] and the fast renal clearance of siRNA and miRNA notably reduce their half-life. Naked miRNAs with unmodified sugar moieties are degraded within seconds by ribonucleases in the biological system [32,33]. Moreover, miRNA cannot pass through the phospholipid bilayers of cell membranes; hence, it becomes impossible for the miRNA to quickly penetrate the endothelial lining of the blood vessels and becomes retained in the organs of blood storage and is thus rapidly cleared by renal excretions [33]. Hence, many investigations have been carried out concerning chemically modifying siRNA and miRNA to increase their stability and bioavailability. These modifications have been found to promote the stability of miRNA by protecting them from degradation [34,35].

There are three moieties in oligonucleotides that can be subjected to chemical modification, namely, the phosphate backbone, ribose, and base. Among the various modifications that have been attempted, the 2-OH modifications of the ribose moiety deserve special attention because it is the preferred target for endonucleases [36]. The incorporation of phosphorothioate on either side of the oligonucleotide strand is reported to increase the deliverability and stability of siRNA [37]. Bioconjugation, where siRNA is conjugated to biomolecules such as antibodies, peptides, carbohydrates, and vitamins, etc. has also been applied to enhance the biological activity and cellular concentration of siRNA. Conjugation with cholesterol has been shown to impart nuclease resistance and multidrug resistance to siRNA therapeutics [38]. The double capacity of aiding siRNA transport in the circulation and facilitating cellular uptake of siRNA is provided by the cholesterol molecule. The cholesterol–siRNA form is immediately fused into lipoprotein complexes such as low-density lipoprotein (LDL) and high-density lipoprotein (HDL) for its uptake into the circulatory system [39]. Inclisiran, an siRNA therapeutic developed and approved for use in patients with hypercholesterolemia, has been conjugated to N-acetylgalactosamine carbohydrates [40].

### 2.4. Innate Immune System Activation

siRNAs are found to induce TNF-α and stimulate IL-6 production by various pathways such as Toll-like receptors (TLRs) [41], retinoic acid-inducible gene-I (RIG)/melanoma differentiation-associated protein 5 (MDA-5) [42], RIG-I-mediated antiviral responses to single-stranded RNA bearing 5′-phosphates, and dsRNA-dependent protein kinase (PKR) [43] pathways. It has been proven that specific sequences on the siRNA sense strand play an important role in its immunostimulatory properties. An optimized design of the siRNA arrangement and structure can likewise assist in preventing siRNAs from being recognized by the intrinsic immune system [44]. Hence, immune recognition of oligonucleotides by signaling pathways can be abrogated by chemical modifications of siRNA. Replacement of 2′-hydroxyluridines in the siRNA with 2′-deoxy or 2′-fluoro or 2′O-methyl motifs could abolish immunostimulation. Substituting the base moiety of oligonucleotides with base analogues can be of great use in reducing the innate immune response associated with such therapeutics [45,46,47]. These challenges are being addressed by the following miRNA and siRNA delivery methods, which are potential novel strategies for future miRNA- and siRNA-based therapeutics (Table 1).

## 3. Modification Strategies to Overcome the Challenges of Nanocarriers

Currently, many formulations of nanocarriers, such as polymeric formulations, liposomes, exosomes, and their combinations have led to improvements in the mechanism and efficacy of miRNA- and siRNA-based therapeutics. Zhang et al. [66] studied the regulation of metastasis in murine breast cancer by developing a system that contained miRNA-10b antagomirs (antagomir-10b) and paclitaxel (PTX, a cytotoxic reagent) using a liposomal delivery system modified with antimicrobial peptide [D]-H_6_L_9_ (D-Lip) that could hinder the 4T1 tumor cells. Recently, a PEG-derived polymeric NP delivery system was used to deliver paclitaxel and siRNA to surviving genes for non-small cell lung cancer (NSCLC). The in vitro and in vivo study results showed that the novel nanoparticle formulation had high drug loading with less toxicity and enhanced antiproliferation effects of PTX on A549 cells [67]. Another study reported that the use of polyethylene glycol-polyethyleneimine (PEG-PEI) nanoparticles for miR-150 transfection overcame the poor transfection efficiency and instability of chronic myeloid leukemia cells [68]. Polymeric NPs have been broadly used for the aerosol delivery of chemotherapeutics, genes, or a combination of the two for lung cancer therapy because of their biocompatibility and respiratory phagocytic mechanisms that allow the drugs’ prolonged exposure to the respiratory system.

The unique physical and chemical properties of poly(amidoamine) PAMAM dendrimers and the many advantages of these polymeric NPs make them an attractive candidate for cancer therapy and diagnosis. This highly branched nano polymeric structure with a multivalent surface and biocompatibility with dendrimers, which provides excellent encapsulation of the drugs, solubilization, and an inherent passive targeting ability, makes them a promising targeted delivery system for siRNA and miRNA therapeutics [69,70].

Oner et al. [71] constructed a modified cationic solid lipid nanoparticle (cSLS) to carry siRNAs targeting the EphA2 receptor tyrosine kinase in prostate cancer and showed improvement in both cellular uptake and gene silencing in the cancer cells, as well as improvements in protecting siRNA against nucleases. Moreover, the cyclodextrin-based carbohydrate polymer combination cell-penetrating siRNA delivery system demonstrated a higher cellular internalization, long biocompatibility, less toxicity, resistance to nuclease activity, increased endosomal escape, and the absence of immunostimulation [72]. These novel strategies in which nanoparticles incorporate miRNA- and siRNa-based delivery systems address some of the issues associated with miRNA- and siRNA-based cancer therapeutics.

## 4. The Importance of Using Nanoparticles for siRNA and miRNA Delivery

Scaling back nanocarriers is a promising technique for targeting fibrotic tumors, such as pancreatic cancer, attributable to improved tissue penetrability. The oligonucleotide structures assume a critical role in the pharmacokinetics of minuscule nanocarriers [73]. Focusing on fibrotic malignant growth, nanocarriers exhibit the best convincing strategies as they enhance the tissue permeability and regeneration. A unit polyion complex (uPIC) utilizing a single oligonucleotide molecule with molecules of two-branched poly(ethylene glycol)-b-poly(l-) (bPEG-PLys) was developed as an oligonucleotide nanocarrier [74]. The results demonstrated that the polyion blending between the oligonucleotide and bPEG-PLys under favorable biological conditions facilitated enhanced blood circulation of the uPICs.

**Figure 3:**
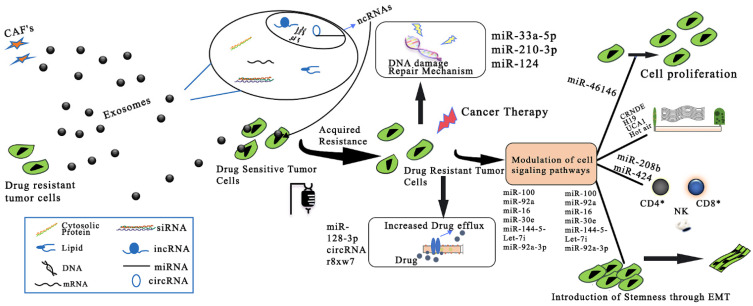
Exosomal ncRNA-related mechanisms implicated in CRC drug resistance. CAFs: cancer-associated fibroblasts; ncRNAs: noncoding RNAs; miRNA: microRNA; circRNA: circular RNA; lncRNA: long noncoding RNA. Adapted and modified from [75] under the Creative Commons license.

The latest mechanism involving RNA DNA and proteins studied for exosome-mediated drug resistance is the transfer of exosomes from the bioactive cargo. The exosomes are transported from either drug-resistant tumor cells or drug-sensitive cancer cells (CAFs). The drug-sensitive cancer cell is communicated with using a drug-resistant phenotype with the help of an exosome-involved mechanism. The general exosomal noncoding (ncRNA) mechanism suggested for drugs approved for CRC is shown in Figure 3. The “collaborative assembly” approach could be achieved by a blended siRNA delivery system using a mix of an electrostatically driven physical assembly and a facile click reaction-mediated chemical assembly. It was demonstrated that the blended siRNA delivery system showed different benefits regarding safety, effectiveness, and adaptability in comparison to the customary methodology. A higher in vivo stability for efficient tumor targeting was obtained when using lipid–polymer hybrid nanoparticles (RSC-HA) with the collaborative assembly approach in comparison to that achieved when using physically assembled lipid–polymer hybrid nanoparticles (RSC/HA) [74].

Laurence et al. developed a secondary amphipathic peptide (CADY) of 20 residues combining aromatic tryptophan and cationic arginine residues to work on cellular uptake of siRNA into testing cell lines. The results showed that CADY is non-toxic and enters cells through a mechanism that is autonomous of the major endosomal pathway. It was suggested that CADY-based innovation will significantly affect the advancement of essential and therapeutic siRNA-based applications with their natural properties [76].

## 5. Important Classes of Nanocarriers Used for siRNA and miRNA Delivery

Nature provides a specific set of materials, architectures, systems, and functions for all basic and complex living organisms [77]. In this section, we center around an examination of the latest progress in the planning, manufacture, and utilization of bioinspired materials used as nanocarriers, especially those with better pharmacokinetic and pharmacodynamic profiles, controlled and sustained release of the drugs, expanded specificity, increased internalization, and intracellular conveyance with lower systematic toxicity. Furthermore, different strategies and optimization techniques for the development of smart bio and bio-hybrid materials are also discussed.

### 5.1. Liposome-Based Nanocarriers

**Figure 4:**
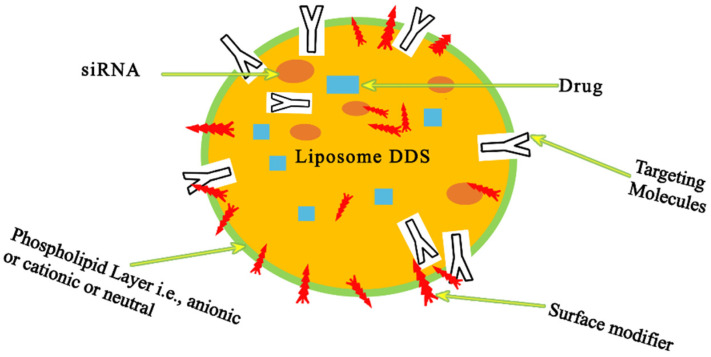
Liposome-based carriers are sphere-shaped vesicles made of synthetic or natural phospholipids. Surface-modifier and -targeting groups can be conjugated to the outer surface. Phospholipids naturally form a bilayer upon aqueous dispersion, with the non-polar tails facing one other and the polar heads facing towards the aqueous phase. Hydrophilic molecules and RNAs are incorporated into the resulting inner core, while hydrophobic molecules are encapsulated in the lipid bilayer. Adapted and modified from [81] under the Creative Commons license.

Chemotherapy can be considered a critical challenge in the therapy of cancer as it is a cycle of delivering significant cytotoxic agents to the malignant growth requiring great precision in managing any unexpected responses due to the drugs [78]. Liposome-based chemotherapeutics can aid in the increase in the therapeutic index of unencapsulated anticancer medications as they can be used for the treatment of malignant growths. The definitive chances of expanded concentrations of the medication to be conveyed to the growth site are partially ascribed to the cytotoxic agents inside the liposomes. Drug pharmacokinetics and biodistribution could be improved with the utilization of drug delivery systems (DDSs) with liposomes as they possess the normal pharmacological properties of commonly utilized chemotherapeutics. These types of nanocarriers are, to a limited extent, an especially appealing DDS because of the simplicity with which they can be created and changed so that they can be used to treat a wide assortment of tumors [79]. As per the perspective of nanotechnology, liposomes are endorsed as the principal drug items for malignant growths and for other restorative applications. A phospholipid bilayer is present in liposomes, and they are characterized as unilamellar or multilamellar microvehicles, as shown in Figure 4. Phospholipids include phosphatidylcholine (PC), sphingomyelin (SM), phosphatidylserine (PS), and phosphatidylethanolamine (PE). They aid in the control of cellular integrity by forming a semi-impermeable hindrance. Phospholipids are composed of hydrocarbon tail (hydrophobic) and polar head (hydrophilic) entities. When presented with water, phospholipids assume a phospholipid bilayer with their hydrophobic tails confronting one another and the hydrophilic heads confronting the water on both ends [80].

M. Saad et al. developed a multifunctional nanocarrier DDS comprising of cationic liposomes, an anticancer drug (doxorubicin (DOX)), and siRNA focused on MRP1 and BCL2 miRNA [33]. The results showed that improved proficiency of chemotherapy using a nanocarrier-based drug system (NDS) cannot be achieved separately without its hybrids. Drug resistance is a daunting challenge to the viable therapy of diseases. For the fundamental co-conveyance of doxorubicin (Dox) therapy for tumor growths, Chen et al. devised several nanoparticle formulations with cationic liposome–polycation–DNA (LPD) and anionic liposome–polycation–DNA (LPD-II) [79]. The research featured a multifunctional nanoparticle with a compelling conveyance property and the capacity to surpass drug resistance in malignant growth effects, likely demonstrating a higher potential for clinical use. LPD nanoparticles containing (*N*,*N*-distearyl-*N*-methyl-*N*-2-(*N*′-arginyl) aminoethyl ammonium chloride) DSAA induced more toxicity in contrast to LPD-II nanoparticles. In order to deliver small interfering RNA (siRNA) to the cancer intravenously, Li et al. devised a self-assembled nanoparticle system that delivers siRNA to the tumor site efficiently [14]. The developed NP system consisted of carrier DNA, siRNA, protamine, and lipids. The post-modification of the system was developed using polyethylene glycol and an anisamide ligand. The accomplishment resulted in considerable cancer growth reduction through the therapy with the designated NPs. Finally, complete hindrance continued for a week upon customizing it with cisplatin. Kang et al. characterized a mitogen-activated protein kinase enzyme (MEK) inhibitor in the lipid layers of *N*′,*N*″-dioleylglutamide-based cationic liposomes (DGL) to test the co-delivery of anticancer small interfering RNA (siRNA) and a chemical MEK inhibitor involving cationic liposomes [82]. This model could serve as an expected methodology for anticancer treatment, such as the use of poly(glucono-δ-lactone) PDGL-intervening co-conveyance of siMcl1 and an MEK inhibitor.

### 5.2. Dendrimer-Based Nanocarriers

These are certain macromolecules that are hyperbranched and monodispersed and are characterized by sub-atomic loads and host properties. These complexes can be interfaced with medication and gene particles by basic encapsulations as they have voided interior gaps with a higher thickness of the substrate functional groups. Dendrimers have some toxicity issues, as indicated by thorough measurements [83].

**Figure 5:**
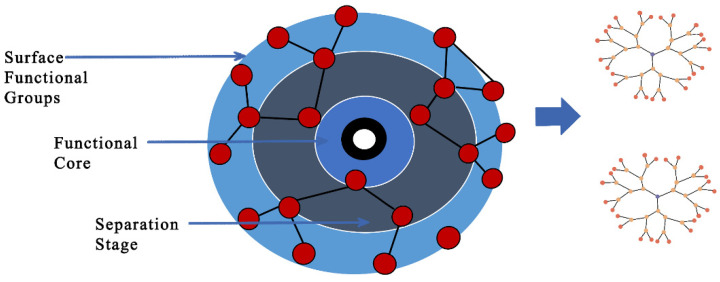
Illustration of dendritic molecular structures with a central core, repeating branches, and terminal reactive functional groups. They can be classified as polymers and hyperbranched polymers with convergent and divergent architectures, depending on their molecular nature weight. Their nanostructure provides dendrimer–drug conjugation via different interactions such as electrostatic and hydrophobic/hydrogen bonds or the capacity for drug encapsulation within the central cavity and/or between the dendrons (branches). Adapted and modified from [87] under the Creative Commons license.

These supramolecular structures were found in the mid-1980s by Donald Tomalia and colleagues [84]. One of the major considerations of dendrimers as a nanocarrier is their size; as they are extensive at the nanoscale, this helps to coordinate with the size of different biomolecules [85]. In fact, their solubility characteristics can fluctuate depending upon the nature of the surface groups, and their size and molecular mass are effectively controllable. Dendrimers are created in an iterative succession of reaction steps, in which each additional iteration prompts a higher generation material. The blend of discrete quantities of functionalities in a single molecule and high local densities of active groups, common for dendritic particles, are crucial suitable material for drug delivery. Dendrimers with different indistinguishable ligands are extremely appealing for pharmacochemists since these structures can display amplified substrate binding [86]. Improved substrate binding starts from either statistical impacts or from cooperativity impacts. The schematic of dendrimer-based nanocarriers is shown in Figure 5 below.

A cationic dendrimer poly(amido amine) (PAMAM) was examined for productive siRNA delivery [88]. With this advent, some polyamine polymers have attracted researchers’ attention as carriers for drug delivery. RNA interference by small interfering RNA (siRNA) remains a prospect in attaining the objective of targeted drug delivery. However, due to its limitations for productive intracellular delivery, the conveyance can be ambitious. A dendrimer complex was agglomerated by Biswas et al. to form a triblock co-polymeric framework [44]. Consolidated treatment with microRNA and chemotherapeutants has emerged as a suitable methodology for improving chemosensitivity. Xiaomin et al. developed a three amphiphilic co-polymer complex containing polylactic acid (PLA) and poly(dimethylaminoethyl methacrylate) (PDMAEMA) [39]. The gene transfection effectiveness and cancer hindrance capacity showed a remarkable reliance on the sub-atomic architecture. The results exhibited that this polymer complex is profoundly encouraging for their joint delivery of genes and therapeutants. The co-delivery of drugs and genes has become an essential technique for treating malignant growths and other infectious diseases. Dong et al. developed a copolymer (PP-PLLD-Arg) comprising a porphyrin (PP) core and arginine-functionalized poly(l-lysine) dendron (PLLD-Arg) arms and observed a photochemical internalization impact on malignant growth disease treatment [79]. A critical reduction in matrix metallopeptidase 9 (MMP-9) protein expression in HNE-1 cells was observed and showed photo-enhanced gene transfection efficiency in vitro. This copolymer also displayed better biocompatibility with blood and lower cytotoxicity effects in contrast to others.

### 5.3. Micelle-Based Nanocarriers and Other Biodegradable Polymers

The effects of poor water solubility, which make them challenging as formulators for parenteral administration and regularly create setbacks for drug advancement, have necessitated the search for new chemical entities. It was observed that most of the dynamic substances (at least 40% or more) that have poor water solubility have been recognized through combinatorial screening programs [89]. Likewise, numerous ineffectively dissolvable drugs that are currently available have not made full use of their true capacity due to the toxicity from the drugs or from their excipients in the formulation. Nanocarriers should have the ability to shield the substance from becoming degraded and to forestall early release so as to accomplish the concurrent conveyance of gene agents and chemotherapeutants. The most detailed instances of co-delivery carriers are micelleplexes comprising amphiphilic block copolymers. Typically, to shape the interior micelle with hydrophilic blocks and to frame the micelle outer shell, the micelle itself collects the required number of blocks. Recently, cationic micelles have been broadly investigated for the delivery of drugs and RNAi-based mixes [7]. These micelle structures are framed by amphiphilic copolymers and are absolutely nanoscopic [90]. Micellar networks have been shown to be the most appropriate for drug delivery applications because of their tunable properties as per the specific region. Some of the critical benefits of nanomicelles include solubilization of ineffective water-soluble molecules, supported delivery, and protecting bioactive molecules from degradation and digestion [91]. Regarding the delivery of different hydrophobic and hydrophilic drugs, cationic micelles have shown tremendous authenticity; however, they face many challenges before reaching clinical trials due to their strength issues.

Gene-related disorders cannot be neglected as they are acquired naturally, and, due to this fact, gene therapy has attracted tremendous consideration due to its enormous potential in treating these disorders. Gene therapy mainly includes the delivery of hereditary materials into target cells and the improvement of a proficient carrier to accomplish this is required for therapeutic effect [92]. Generally, to surpass the intricacy of cancers, a mix of restorative drugs would be a promising method by which to induce an impact through therapy. Gene transfer to mammalian cells mediated by small molecular amphiphile–polymer conjugates, such as bile acid–polyethylenimine (BA-PEI), was found to be effective by Chae et al. [88]. To improvise the conveyance of macromolecular therapeutics, this new methodology using polymeric biomimetics can be applied. Cao et al. agglomerated the biodegradable nano carriers (PEI-PCL) of poly(ε-caprolactone) (PCL) and direct poly(ethylene imine) (PEI) for the co-delivery of B-cell lymphoma/leukemia-2 BCL-2 siRNA and doxorubicin (DOX) [93]. The folate-designated conveyance of BCL-2 siRNA brought about more significant gene suppression for both the BCL-2 miRNA and protein expression levels, leading to an incitement of malignant growth cell apoptosis and enhancement in the restorative viability of the co-administered DOX. Zhu et al. prepared cationic micelles with biodegradable PCL from PDMAEMA-PCL-PDMAEMA triblock copolymers and applied the conveyance of siRNA and paclitaxel into disease cells [94]. Higher drug efficacy was reported in micelle 1 stacked with paclitaxel than in free paclitaxel in PC3 cells due to enhanced cell take-up. For the co-delivery, Zhang et al. blended a poly(ethyleneimine)-poly(γ-cholesterol-L-glutamate) (PEI-PCHLG) copolymer [95]. The complexes formed from dual-functional PEI-poly(γ-cholesterol-l-glutamate) (PEI-PCHLG) copolymers are definitive approaches, being additionally evolved as significant drug carriers on account of their resistance to dilution in the circulation. Finally, after creating novel double functional PEI-poly(γ-cholesterol-l-glutamate) (PEI-PCHLG) copolymers, a progression of PEI-PCHLG (PEI-1, PEI-2, PEI-3, and PEI-4) with different PEI percentages and molecular weights were effectively incorporated. Better encapsulation proficiency and higher stacking of DTX in PEI-PCHLG (PEI-4) micelles showed that the micelles could effectively completely consolidate hydrophobic drugs.

The usage of NPs derived from biodegradable entities has been vigorously researched because of their higher efficiency in the release of drugs within the cytoplasm and because their complexes are comparatively easily manufactured with relevant functionalization for the targeting molecules [52]. Currently, amongst the biodegradable polymers, polycaprolactone (PCL) and PLGA are the most used for miRNA delivery. The non-cytotoxic and biocompatible polymer PLGA undergoes hydrolytic degradation to form lactic and glycolic acid monomers. Usually, the degradation rate is slower for these polymers but by changing the proportions between the lactic and glycolic acid monomers, their degradation rate can be tuned to be faster. Polymers with a higher glycolide content and a lower molecular weight are always hydrophilic with faster degradation rates, while polymers with a smaller glycolide content and a higher molecular weight take longer to degrade. Contingent upon the chemical structure of the molecule, every polymer’s degradation rate is different. For instance, as contrasted with PLA, PGA has a faster degradation rate. As a copolymer of PLA and PGA, PLGA manifests a degradation rate that is intermediate between them.

PCL has also attracted significant attention for the development of biocompatible NPs for protein and drug delivery. NPs made out of PCL are extremely favorable due to their high colloidal strength, faster cell take-up, higher biocompatibility, and controlled delivery of their drug carriers [96]. To deliver miR-200c and docataxel, Li et al. developed a PEG–peptide–PCL NPs complex using the double emulsion method [97]. To demonstrate a faster delivery after the NPs’ internalization effect on the tumor cells, a suitable peptide with a gelatinase complex was selected. Yang et al. integrated biodegradable charged polyester-based vectors (BCPVs) for the co-delivery of K-ras and Notch1 (siRNA) into a pancreatic cell line of MiaPaCa-2 to overcome the resistance created by a drug from gemcitabine (GEM) [98].

## 6. Advantages of Nanocarriers in miRNA Therapeutics

**Figure 6:**
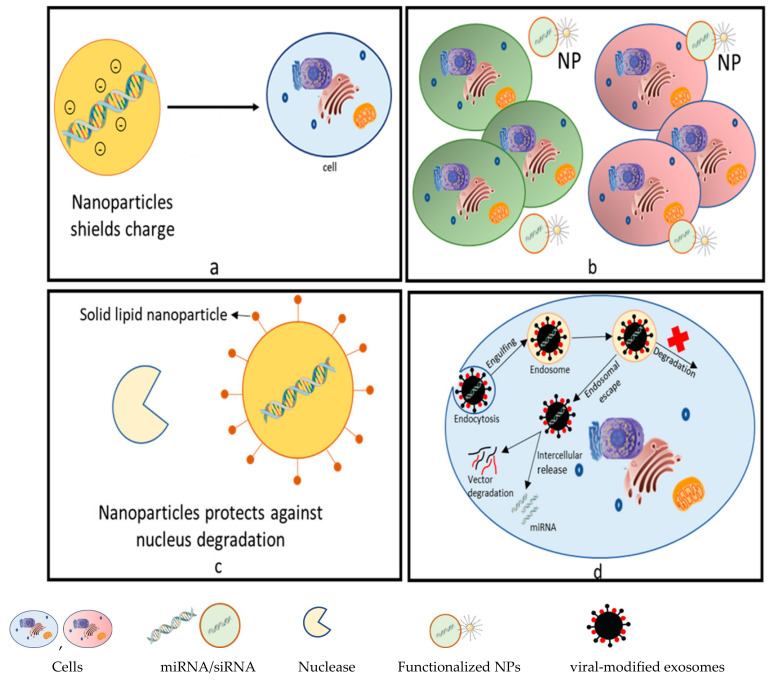
Advantages of nanoparticles. (**a**) Nanoparticles (NPs) encapsulate miRNA/SiRNA, thus shielding the charges and improving the penetration/uptake of cells. (**b**) Functionalized NPs (mesoporous silicon) enable controlled and cell-specific miRNA/siRNA delivery. (**c**) Solid lipid nanoparticles (SLN) protect miRNA/siRNA from degradation and provide high stability. (**d**) Virus-modified exosome (combination of NPs) enables siRNA/miRNA delivery into target cells, improving target specificity and endosomal escape.

**Figure 7:**
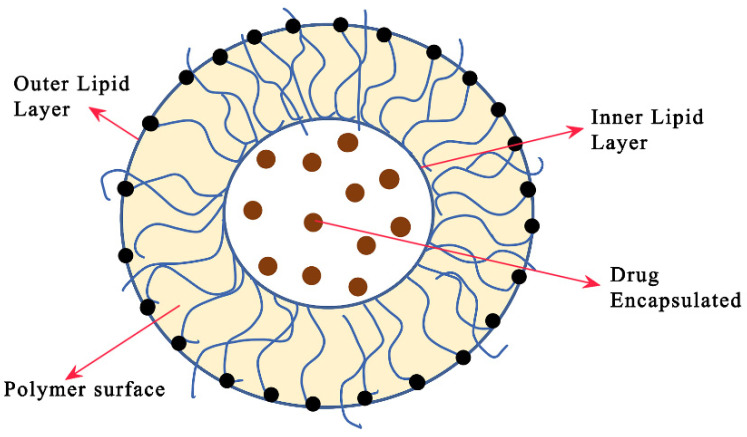
Illustration of a bilayer lipid-based nanocarrier with encapsulated drugs in the core; the self-assembled supramolecular architecture is shown. The solid lipid matrix encapsulates bioactive components, particularly lipophilic molecules, and releases them gradually over time. Lipid polymer nanoparticles typically have spherical particles and sizes in the range of 10 to 1000 nm. There are several forms of lipid-based nanocarriers (liposomes and niosomes) reported in reference [81] for drug delivery.

Since the stability of naked miRNAs is low, and since they are susceptible to rapid degradability, the efficient and safe delivery of miRNA is potentially a novel therapeutic modality. Moreover, the obsolete inactivating procedures are not suitable for human disease-related applications. Improved therapeutic strategies can be attenuated by the encapsulation of the miRNA into nanoparticles. These complexes can overcome the present drawbacks and result in optimized efficiency, protecting the oligonucleotides from serum RNase degradations. Unfavorable immune stimulation, undesirable off-target or on-target effects, and negatively charged groups of miRNAs are some of the associated drawbacks of the naked delivery of miRNAs that are overloaded using nanoparticle carriers. The shielding charge groups and the uptake of the cells are activated by the encapsulation of miRNAs by NPs. The functionalization of NPs with cell-specific ligands can reduce the off-target effects by permitting nanocarriers to transport miRNAs to the target cells. This will allow NPs for controlled miRNA release, avoiding excessive activation of multiple gene targets. Usually, payload degradation in vivo is prevented and, in this way, the NPS can enhance the half-life of miRNA. Moreover, undesired immune system responses are prevented by the stealth coating of NPs that enables their clearance by the reticuloendothelial system (RES) [52]. Figure 6 represents the advantages of using NPs for miRNA/siRNA delivery. Figure 7 depicts an illustration of lipid nanoparticles in miRNA therapeutics.

Recent studies have shown different types of nanocarriers with distinguished properties that can be used for miRNA delivery, such as polymeric nanoparticles, dendrimer-based nanocarriers, micelle-based nanocarriers, liposome-based nanocarriers, and inorganic nanoparticles and organic nanoparticles. The miRNA is transferred inside the target cells as the liposomes easily pass through the cell membrane. The selectivity of the liposome can be enhanced by surface modification of the liposomes. Cationic liposomes are safe, non-pathogenic, non-immunogenic, and comparatively easy to prepare. Due to their lower stability and nonspecific binding, affinity toward serum proteins employing the nanocarrier for miRNA delivery of the liposome is restricted [99]. Endosomal escape can aid the delivery of miRNA delivery due to the amine branch acting as a proton sponge in the dendrimers. Micellar particles are suitable for drug delivery, and the application of hydrophobic and hydrophilic drugs can be advantageous due to their modifiable property of the region [100,101].

Recent research has shown the potential of polymeric nanoparticles in acting as drug carriers of RNAi-based therapeutics. Another advantage of NPs is that they allow selective targeting of specific neurons by crossing the blood–brain barrier. NP-mediated drug delivery to the brain can provide better insights into miRNA-based neurodegenerative disease treatments. Solid lipid nanoparticles (SLN) have recently gained much attention as delivery vehicles for siRNA because of their advantages such as stability, safety, and ease of production [102].

Advances have been made in the development of novel polymeric nanomaterials, which include natural and synthetic ones, as non-viral delivery systems for RNA. Cyclodextrin, a naturally occurring oligosaccharide of bacterial origin, has been well studied as a delivery system, and such delivery systems for cancer therapy have been reviewed by Mousazadeh et al. [72].

Some of the non-viral vectors extensively used for the delivery of miRNA include inorganic materials, such as gold nanoparticles (AuNPs), Fe_3_O_4_, mesoporous silicon, and graphene oxide-mediated nanoparticles. The honeycomb structure provides a high specific surface area, and this allows the GO for the high-density loading of drugs through electrostatic bonding [103]. Mesoporous silica nanoparticles (MSNs) have numerous advantages over other NPs, such as larger surface area, optimum pore volume, easy surface adjustment, thermal stability, and biocompatibility [104]. The potentiality of chemotherapeutic agents can be well augmented using siRNA, and hence the design of novel drugs, i.e., cancer drugs in combination with siRNA, has become a trending topic. Nanoparticle platforms incorporating miRNA and chemotherapeutic drugs have been developed for Kirsten rat sarcoma virus (KRAS)/P53 targeting in non-small cell lung cancer [105].

## 7. Nanocarrier Customizations for the Delivery of Gene Agents for Cancer Therapy

The premise of this section is to uncover the significant innovations relating to drug delivery systems (DDSs) and their expected effects through a broad examination of the utilization of these frameworks. Drug targeting through nano carriers in cancer therapy is a highly intricate process that requires innovative DDSs. In the process of drug delivery, the drug is first absorbed into the body, then released into blood, and finally reaches the tumor site. Due to insufficient delivery, most of the agents end up settling in many tissues. Smart drug targeting allows the drug to enter in appropriate amounts and can prevent the agglomeration of drugs in unwanted tissues, thus ensuring a destined efficacy in the targeted environment.

Sepantafar et al. has discussed the use of a hydrogel drug delivery system (DDS). The advantages of this novel delivery system are improvements in chemotherapy results; increased gene therapy efficacy by increasing the drug’s half-life; and facilitating controlled and adjustable drug release, which would subsequently decrease nontargeted exposure [106]. Huang et al. designed a smart nano drug delivery system by masking (doxorubicin and indocyanine green-loaded superparamagnetic iron oxide) SPIO@DOX-ICG nanoparticles within the cancerous cell membrane to augment the efficacy of cancer therapy [89]. The nano-system accumulated precisely in the tumor region and achieved interdependent anti-cancer effects without any toxicological issues. Gao et al. summarized the development of an acid-responsive polymeric DDS with a defined investigation of the acidic microenvironment within the tumor. Using acid-responsive chemical bonds for the fabrication of the DDS, the defined systems were responsible for acting against physiological barriers by surface charge conversion, nanostructure dissociation, and ligand presentation. Ozlu et al. prepared a hybrid diffusion-controlled DDS with polyethylene glycol-conjugated melanin nanoparticles (MNPs) with a load uptake of doxorubicin (DOX) in discrete concentrations [107]. The drug molecules exit through a small perforation due to excessive pressure caused by the volumetric increase. The devised black phosphorus nanosheet polydopamine Ag NP dSIS-BPNs-PDA@Ag complex was able to constrain the proliferation of human breast cancer cells due to the prolonged drug release with a sufficient therapeutic concentration. Zhen et al. reported on the development of semiconducting photothermal nanoagonists with capsaicin (Cap) as the photothermal nanocarrier and the agonist for the multiple activation of transient receptor potential channels (TRPV_1_) responsible for the apoptosis of cancer cells [108]. This remotely controlled DDS assures a high concentration of TRPV_1_ against the tumor site, thus allowing for an efficient systematic dosage. Su et al. established a 3D ECM scaffold from porcine small intestine that can be employed to treat trauma-related bacterial infections for post-operative skin cancer therapy [109]. The bioactive composite scaffold black phosphorus nanosheet polydopamine and Ag nanoparticles (gal) demonstrated higher bactericidal effects against *E. coli* and *S. aureus* and also effectively curbed tumor recurrence through the generation of hyperthermia and hydroxyl radicals.

Recent studies have shown that a magnetic hydrogel with higher magnetic responsiveness can release substantial concentrations of drugs from the hydrogel network. Paulino et al. devised a natural polymer-based hydrogel with 50nm diameter magnetite FeO: Fe_2_O_3_ NPs [110]. This development proportionately substantiated the efficacy of the polysaccharide-modifying process and exhibited improvements in mechanical and thermal resistance in the presence of magnetite FeO: Fe_2_O_3_ NPs. Jahanban et al. designed a pH-responsive magnetic natural hydrogel with Fe_3_O_4_ magnetic nanoparticles on an alginate–gelatin matrix [111]. Due to the presence of carboxylic acid groups in the drug delivery system, the Alg-Gel/Fe_3_O_4_-Dox complex showed pH-dependent drug release behavior. and it also determined suitable magnetic properties for smart drug delivery uses. Furthermore, exosomes have emerged as appropriated therapeutic agents and delivery platforms due to their endogenous characteristics and distinctive biological properties. Conventional strategies have been determined for the expansion of the production and assembly of exosome-like nanovesicles obtained from producer cells. Exosomes develop the cell–cell interactions and indicate systematic ways for the release of different therapeutic agents to the target cells [112]. To accomplish a dynamic focus on targeting drug delivery systems, Yan et al. set up a biomimetic exosome (Exo) with dexamethasone sodium phosphate (Dex) nanoparticles (Exo/Dex) [113]. The sample substrate was altered with folic acid (FA)–polyethylene glycol (PEG)–cholesterol (Chol). To improve the therapeutic impact of glucocorticoids (GCs) against RA, utilizing the exosome as a nanocarrier remains one of the most promising procedures. Kim et al. developed various procedures for inserting exosomes released by macrophages with paclitaxel (exoPTX), allowing exosome-encapsulated paclitaxel to overcome multidrug resistance (MDR) in cancer cells [114]. The exosomal membrane was reformed under the process of sonication and was determined to be viable in delivering a higher loading efficiency with a sustained release profile. Furthermore, this can be extended as an innovative channel for the distribution of diverse chemotherapeutics for diagnosis. Despite significant interventions in the field of exosome drug delivery approaches, further development and validation of these DDSs must be carried out.

Nanoscale particles are aggregated in growth tissue, as contrasted with typical tissue, due to the impact of enhanced permeability and retention (EPR). Typically, nanoparticles within the range of 20–100 nm are recommended so as to ensure a considerably enhanced permeability and retention (EPR) impact. The soundness and biodistribution of the nanoparticles inside the body are decided by an additional significant variable called surface charge [115]. Cationic and anionic liposomes, in contrast to independently charged liposomes, actuate the supplement framework through various pathways. In order to deliver anti-cancer drugs more efficiently to tumor sites by enhancing blood compatibility, Xiao et al. presumed that a partial negative charge might be introduced to the NPs [116]. These investigations recommend that the nanoparticle surface properties should be upgraded for the surface charge to achieve improved intertumoral delivery.

Triantennary N-acetylgalactosamine, referred to as GalNAc, is conjugated to inclisiran, a chemically modified siRNA, to form the complete siRNA complex. Inclisiran is produced using one 2′-deoxy, eleven 2′-fluoro, and 32 2′-O-methyl altered nucleotides [117]. Usually, the ends of the longer passenger strands are functionalized with triantennary GalNAc, and phosphorothioates are used in order to modify the termina; ends of the duplex. The protein production is prevented as soon as inclisiran is delivered through GalNac through hepatocytes. The strand of the complex enters and hybridizes to proprotein convertase subtilisin/kexin type 9 PCSK9 mRNA and finally splits it. N-acetylgalactosamine (Gal-NAC)-designated dynamic polyconjugate DPCs were used by Marija et al. to show the productive delivery of an apolipoprotein B (ApoB) siRNA into hepatocytes [118]. These results were compared to liposome–liposome-interceded ApoB knockdown in the liver. The advantage of using a similar ApoB siRNA complex is that it lasts for longer cycles with a smaller quantity required for ApoB knockdown. One of the remarkable elements of DPCs is their compact size (10–20 nm), which is far smaller in comparison to most liposomes or polyplexes with sizes of around 80–200 nm [119]. The DPCs’ compact size might permit the particle to enter deep into tissues to arrive at cells that are far away from blood vessels, while most other particulate-based DDSs may only enter cells near the blood vessels because of their bigger particle size. This subsequently restricts the delivery effectiveness and the potential utility of these DDSs for applications other than in tumor growth. Moreover, preclinical and clinical examinations directed by Alnylam Pharmaceuticals showed that free siRNAs, focusing on transthyretin TTR formed on a triantennary or trivalent ligand containing monovalent subunits of N-acetylgalactosamine (Gal-NAc) subunits, were dynamically active upon subcutaneous dosages as low as 1 mg/kg and were supported with assisted gene silencing with a repetitive dosage for a span of 9 months. Unconjugated siRNAs are also less harmful, even at doses as high as 300 mg/kg in animal models.

Over the last twenty years, the utilization of nanodelivery frameworks has arisen as a viable method for potentiating restorative properties against diseases [120]. Numerous approaches are currently focused on the planning of optimal specific interactions for an exceptional collaboration with malignant growth cells to work on the remedial viability of customary disease treatment [121]. Lipid–polymer hybrid nanoparticles (LPNs), newer-generation delivery therapeutics, were developed to address the limitations of polymeric nanoparticles and liposomes. The qualities of both polymeric nanoparticles and liposomes are combined in LPNs. LPNs consist of an inner lipid layer, a polymeric core, and an outer lipid–PEG layer. During the LPN development, there is excessive leakage of the encapsulated content. To avoid these circumstances, the inner lipid layer acts as molecular boundary to decrease its overflow. The layer also aids in slowing down the rate of degradation as slower degradation profiles are required for specific applications. This is achieved by controlling the rate of the diffusion of water, consequently leading to a sustained release from the complex.

Tooth et al. [122] used the sonication method and developed a faster nanoprecipitation process by providing a higher and homogeneous energy input. This process allowed a faster gathering of the LPNs, resulting in a twentyfold increase in the efficiency as contrasted with the customary nanoprecipitation technique. Valencia et al. researched and developed a microchannel through nanoprecipitation to explore the homogeneity obtained in the size of the LPNs [123]. The development enabled the optimal blending of lipid/polymer, and the control of the particles could be exactly mediated. Hypothetically, blending of the fluid phase containing the lipid and the natural phase containing the polymer at the microscale prompted homogenous polymer nucleation, allowing the development of LPNs with a profound homogeneous size.

DDSs tend to act against the tumor environment based on their controlled release profile. Usually, sustained release is particularly important for tissue engineering and regenerative medicine as the release of bioactive molecules is synchronized with the duration of the regenerative process. Hence, it is imperative to design and develop efficient bioinspired materials for drug delivery.

## 8. Application of Nanocarriers

Due to various compositions and biological properties, nanoparticle carriers are highly suitable for delivering therapeutic miRNA in a controlled and cell/tissue-specific manner. The liposome- and PAMAM dendrimer (polymer)-based nanoparticles are used for an enhanced permeability and retention (EPR) effect in cancer therapy [124]. It was observed in the hindlimb ischemia model that nanosized miRNA-126-loaded bubble liposomes (polyethylene glycol-modified liposomes) combined with ultrasound promoted the accessibility of deep tissues and improved blood circulation [125]. Poly(lactide-co-glycolide) (PLGA) has been widely used in clinics because of its excellent drug-releasing properties inside the cells [126]. The significant advances in non-viral nanoparticle-based delivery systems for miRNA- and siRNA-based therapeutic approaches in cancer are summarized in Table 2.

## 9. Opportunities in miRNA Therapeutics

The rapid developments that have been made in understanding the functions of miRNA and siRNA have led to the initiation of early-phase clinical trials testing the safety and efficacy of various miRNA- and siRNA-based therapeutics. Researchers are focusing on siRNA inhibition/suppression and miRNA replacement therapies. On the one hand, miRNA suppression therapy includes anti-miRs (antagomirs, antisense oligonucleotide (ASO), modified ASOs, and LNA-based ASOs), miRNA sponges, and small-molecule inhibitors. On the other hand, miRNA replacement therapy consists of its precursors, agomirs, mimics, and expression vectors.

Miravirsen is a locked nucleic acid (LNA)-phosphorothioate modified oligonucleotide (SPC3649) and is the world’s first anti-miRNA drug candidate that has entered clinical trials [141]. Miravirsen was used to treat hepatitis C virus (HCV) infection that targets miR-122 in phase II clinical trials in 2017. miRNA replacement therapy has also shown some promise with MRX34, which is a liposome-based miR-34 mimic and is currently in phase I clinical trials for advanced hepatocellular carcinoma patients [142]. At present, several clinical trials have been started for miRNA- and siRNA-based therapeutics (Table 3).

Two important phase I trials evaluating approaches based on miRNAs complexed with nanoparticles to treat cancer have been completed. MRX34, a liposomal-based miR-34 mimic, was tested in 85 patients with advanced solid malignancies following intravenous administration [3]. Although the trial was closed early due to severe immune-mediated adverse events at higher doses in four patients, MRX34 was found to be reasonably well tolerated at lower doses following dexamethasone premedication. Of the 66 patients in whom clinical responses could be evaluated, 3 (5%) had a partial response and 16 (24%) had stable disease. Similar findings were seen in the phase 1 trial of miRNA mimics delivered by targeted bacterial minicells (TargomiRs), miR-16 mimics packaged in bacterially derived nanocells, which were administered intravenously to 26 patients with progressive mesothelioma [143]. Immune-mediated adverse effects were again observed at higher doses but were well controlled at lower doses following dexamethasone premedication. Of the 22 patients in this study whose clinical response was evaluated, 1 (5%) had a partial response and 15 (68%) demonstrated stable disease. These important studies highlight the potential clinical efficacy of these miRNA-based therapies. It is important to note that in these trials, the miRs were tested only as a monotherapy. It is most likely that the greatest therapeutic effect will be seen with a combination of the miRs and chemotherapy or targeted agents.

More recently, with further modification of the nanoparticle technology, even more impressive results have been observed with RNA-based therapeutics in non-malignant diseases. This miR has been shown to be required for the propagation of the hepatitis C virus (HCV) in the liver. In a phase II clinical trial, Miravirsen was shown to be very well tolerated following subcutaneous injection and resulted in a dose-dependent reduction in serum HCV RNA levels at all three dose ranges tested in 36 patients [144]. Most notably, Patisiran, a liposomal based siRNA targeting transthyretin, was recently approved by the FDA and European Commission for the treatment of hereditary transthyretin-mediated amyloidosis in adults. This landmark event marks the breakthrough of RNA-based therapeutics from the laboratory to the clinic and will likely lead to the adoption of these approaches for cancer treatment soon.

## 10. Conclusions and Future Perspectives

Cancer remains a major cause of mortality worldwide. Despite years of research, therapies are still in the process of advancement, but the accessibility of effective and pristine targeted therapeutics is very limited. A more basic variable that assumes a fundamental part in effectively managing this disease is a protected and target-specific DDS. Even though different restoratively dynamic moieties have aided in repressing malignant growth, their downsides include lower bioavailability and toxic immune responses. Different nanoparticle-based delivery systems such as liposome-, dendrimer-, and micelle-based systems, have been evaluated and their efficacy and viability in vitro and in vivo is reviewed above.

Even with their enormous potential to achieve robust target gene silencing, the low cellular take-up of unmodified siRNAs and miRNAs remains a significant limitation to the effective utilization of these molecules due to their inability to infiltrate cells with high efficacy. It has been shown that encapsulated siRNA and miRNA nanoparticles can effectively enter cells and exit from endosomes, ensuring explicit target gene silencing in diseased cells. Hence, siRNA and miRNA systems should be complexed or formed with a proper delivery system. In fact, various techniques, including the use of biocompatible and adaptable carriers with modified siRNAs and miRNAs, have brought about potential RNAi-based drugs.

Despite critical advances in in vivo delivery systems, there are still several difficulties and boundaries that need to be overcome to accomplish the most optimized formulations. Currently, the FDA has endorsed only a few groups of nanoparticle-based siRNA and miRNA delivery systems, which are undergoing preliminary clinical testing. Future investigations should be extensively focused on the in vivo applications of the different delivery systems that limit harmful immune responses and cytotoxic issues. In addition, investigations evaluating the intratumoral, instead of intravenous, delivery of RNAi-based drugs should be strongly considered. Direct intratumoral injection offers several potential advantages over systemic drug delivery, including the avoidance of rapid clearance, the achievement of higher intratumoral concentrations by as much as 10–30-fold, and decreased systemic toxicities and immune responses. The clinical feasibility and efficacy of direct intratumoral injection of chemotherapeutic agents has been demonstrated in multiple malignancies including lung, brain, and neck malignancies. Complexing siRNAs with miRs and various conveyance frameworks for direct intratumoral injection has proven effective in several pre-clinical models [145]. With these necessary improvements in mind, the development of biocompatible and biodegradable nanoparticle-based drug delivery systems for the clinical use of RNAi-based disease therapeutics is crucial and should be fostered innovatively to fight cancer.

## Figures and Tables

**Table 1:** A comparison of the potential delivery strategies for miRNA- and siRNA-based therapeutics.

Delivery Method	Viral	Liposome	Conjugates	Extracellular Vesicles	Polymers	Nanoparticles
**Widely used vectors**	Retrovirus, lentivirus, adenovirus, adeno-associated virus	Forms lipoplex by the interaction of cationic lipids	Receptor binding molecules bind directly to nucleic acids: aptamers, multi-functional peptides [48]	Exosomes, microvesicles, platelets, apoptotic bodies	Polyethyleneimine (PEI), poly (lactide-co-glycolide), poly (amidoamine), dendrimers/cell-penetrating peptides [49,50]	AuNPs, mesoporous silicone, graphene oxide, and Fe_3_O_4_-mediated NPs [50,51,52]
**Advantages**	High transfection efficiency [50,51,52]	Biodegradable, biocompatible Reduces the toxicity High affinity with cell membrane Increased efficacy and therapeutic index [53]	Less toxicity, higher stability, selective targeting, high intracellular delivery efficiency [33,54]	Low cytotoxicity and negligible antigenicity, naturally present in body fluids, ability to cross blood–brain barriers [50]	Natural and synthetic polymers: Biocompatible. Natural polymers: less toxicity, biodegradable [49,50]	High stability in vivo, free of microbial attack [50,51]
**Disadvantages**	Low loading capacity, high toxicity, strong immunogenicity, mutation [50,51,52]	Limited storage settings Poor stability Short half-life Low solubility [53]	Endosomal entrapment [33,54]	Low drug loading capacity, rapid clearance from blood [50]	Natural polymer: Highly branched structures, complicated [49,50] extraction method Synthetic Polymer: High toxicity, poorly biodegradable [49,50]	Inorganic material: weak interaction between the carrier and nucleic acids [48,50]
**siRNA examples**	Adeno-associated virus vector for p53 siRNA delivery into HeLa S3 cells [55]	Core-shell lipoplexes encapsulated c-Myc-targeting siRNA for glioblastoma treatment [56]	Cholesterol-tethered EpCAM-targeting RNA aptamer for cancer stem cell-targeted gene delivery [57]	Artificial platelets for efficient delivery of siRNA targeting Pcsk9 transcription in the liver [58]	PEI-cholesterol-polyethylene glycol, Dendrimers [59]	Functionalized gold nanorod-based TFEB-siRNA autophagy for osteosarcoma [60]
**miRNA Examples**	Lentivirus-miR-199a inhibition of HCC cell proliferation [52]	miR-7 encapsulated with cationic liposome against EGFR-TKI-resistant lung cancer cells [61]	Lipid NP-mediated anti-miR-17 family suppression of Hep3B tumor growth [62]	Brain metastasis cancer cell-derived EV-miR-181c promotes brain metastasis and destruction of the blood–brain barrier [63]	LbL-PLGA NPs carries miR-34a cargo for suppression of target gene and reduced triple-negative breast cancer cell proliferation [64]	Anti-miR-155-loaded modified mesoporous silica NPs (MSNs-anti-miR-155@PDA-Apt) for colorectal cancer [65]

**Table 2:** Non-viral nanoparticle-based delivery systems for miRNA- and siRNA-based therapeutic approaches in cancer.

Delivery System	Target	Disease	miRNA/siRNA	Ref.
Gold NPs	Mcl-1	HeLa cell cancer	miR-29b	[126,127]
	Plk1	Breast cancer	siPlk1/Ap-Cs	[128]
Silica-based NPs	MYCN	Neuroblastoma	miR-34a	[129]
	MDR1	HeLa-RDB	NH2-MSN-siRNA with chitosan coat	[130]
PAMAM-dendrimer	miR-21	Glioblastoma	as-miR-21	[131,132]
	Hsp27	Prostate cancer	TEA core-PAMAM dendrimer-siRNA	[133]
PLGA NPs	SHIP1	Lymphoma/leukemia	miR-155	[134]
	MDR1, Bcl2	Ovarian cancer	siRNA-loaded PLGA NPs	[135]
Chitosan NPs	Survivin	Breast cancer	PEG-chitosan-siRNA	[136]
Cationic lipoplexes-lipid-based NPs	PKCε, CDK6, HIF1-β	Head and neck squamous cell carcinoma (HNSCC)	miR107	[137]
	EGFR	Brain tumor	T7-LPC-siRNA Nps	[138]
Liposomes	Slug	Triple-negative breast cancer	miR-203	[139]
	MDR1	Squamous carcinoma	2x3-DOPE/FC liposome siRNA	[140]

**Table 3:** Clinical trials that have been started for miRNA- and siRNA-based therapeutics. Data based on the clinical trial (https://clinicaltrials.gov/).

Drug Name and miRNA Target	Disease	Category	Company	Clinical Trial Details (Identifier) and Status
MRX34, miR-34a	Hepatocellular carcinoma	Mimic	MiRNA Therapeutics	NCT01829971, Phase I, Terminated
MesomiR-1, miR-16	Mesothelioma and non-small cell lung cancer	TargomiRs (mimic)	EnGeneIC Limited	NCT02369198, Phase I, Completed
Cobomarsen/MRG-106, miR-155	Cutaneous T-cell lymphoma/mycosis fungoides	LNA modified antisense inhibitor	miRagen Therapeutics	NCT03713320, Phase II, Terminated
Remlarsen/MRG-201, miR-29	Keloid	Mimic (cholesterol-conjugated miR duplex)	miRagen Therapeutics	NCT03601052, Phase II, Completed
AZD4076/RG-125, miR-103/107	Type 2 diabetes mellitus and non-alcoholic fatty liver disease	GalNAC-conjugated Anti-miR	AstraZeneca	NCT02826525, Phase I, Completed
CALAA-01/M2 subunit of R2	Solid tumor	Cyclodextrin NPs	Calando Pharmaceuticals	NCT00689065, Phase I, Terminated
EphA2-targeting DOPC-encapsulated siRNA/EphA2	Advanced solid tumor with liver metastases	Neutral liposomes	M D Anderson Cancer Center, Houston	NCT01591356, Phase I, Completed
TKM-080301/PLK-1	Primary and secondary liver cancer	Lipid nanoparticles	National Cancer Institute (NCI)	NCT01437007, Phase I, Completed

## Data Availability

Not applicable.
