# Correction: Zare et al. Encapsulation of miRNA and siRNA into Nanomaterials for Cancer Therapeutics. *Pharmaceutics* 2022, *14*, 1620

**DOI:** 10.3390/pharmaceutics15010279

**Published:** 2023-01-13

**Authors:** Mina Zare, Rakesh Pemmada, Maya Madhavan, Aswathy Shailaja, Seeram Ramakrishna, Sumodan Padikkala Kandiyil, James M. Donahue, Vinoy Thomas

**Affiliations:** 1 Center for Nanotechnology and Sustainability, Department of Mechanical Engineering, National University of Singapore, Singapore 117581, Singapore; 2 Department of Food and Nutrition, University of Helsinki, 00014 Helsinki, Finland; 3 Departments of Materials Science and Engineering, Biomedical Engineering, University of Alabama at Birmingham (UAB), Birmingham, AL 35294, USA; 4 Department of Biochemistry, Government College for Women, Thiruvananthapuram 695014, India; 5 Department of Pediatrics, Duke University School of Medicine, Durham, NC 27710, USA; 6 Post Graduate Department of Zoology, Government College, Madappally 673102, India; 7 School of Medicine, University of Alabama at Birmingham, Birmingham, AL 35294, USA; 8 Center for Nanoscale Materials and Biointegration (CNMB), Center for Clinical and Translational Science (CCTS), University of Alabama at Birmingham (UAB), Birmingham, AL 35294, USA

## Enhancement in Figure:

In the original publication [1], the figures were not sharp enough. The enhanced Figure 1, Figure 3, Figure 4, Figure 5, and Figure 7 appear below. The authors state that the scientific conclusions are unaffected. This correction was approved by the Academic Editor. The original publication has also been updated.

## Figures and Tables

**Figure 1:**
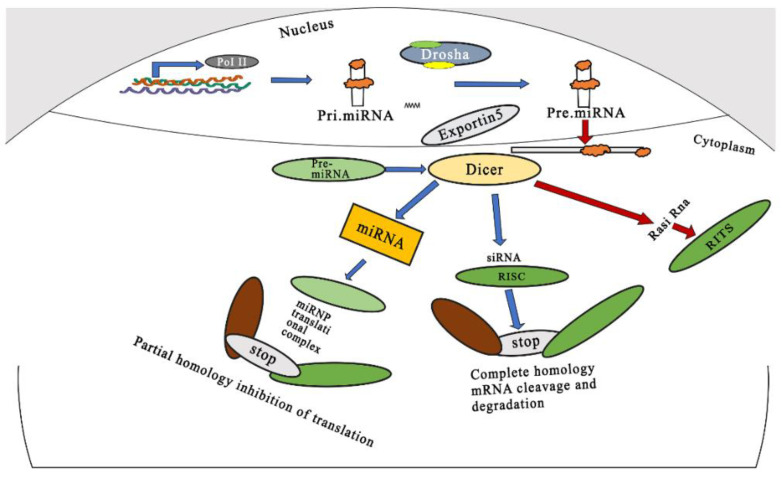
Schematic representation of mechanism of gene silencing by miRNAs and siRNAs. Adapted and modified from [12] under the Creative Commons license.

**Figure 3:**
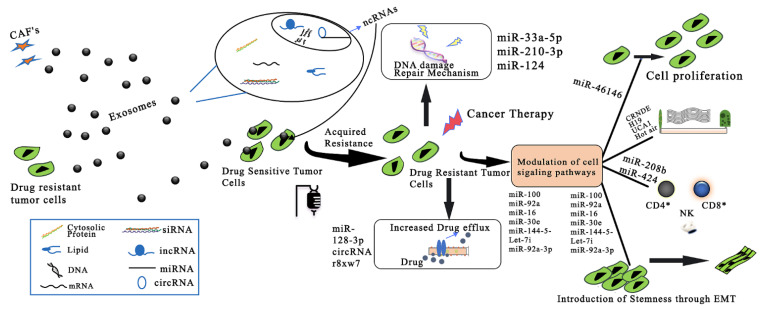
Exosomal ncRNA-related mechanisms implicated in CRC drug resistance. CAFs: cancer-associated fibroblasts; ncRNAs: noncoding RNAs; miRNA: microRNA; circRNA: circular RNA; lncRNA: long noncoding RNA. Adapted and modified from [75] under the Creative Commons license.

**Figure 4:**
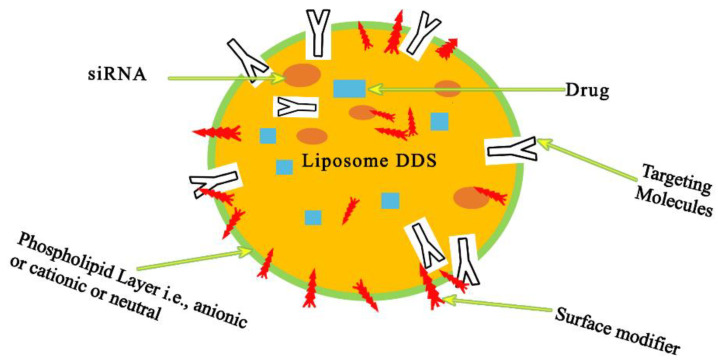
Liposome-based carriers are sphere-shaped vesicles made of synthetic or natural phospholipids. Surface-modifier and -targeting groups can be conjugated to the outer surface. Phospholipids naturally form a bilayer upon aqueous dispersion, with the non-polar tails facing one other and the polar heads facing towards the aqueous phase. Hydrophilic molecules and RNAs are incorporated into the resulting inner core, while hydrophobic molecules are encapsulated in the lipid bilayer. Adapted and modified from [81] under the Creative Commons license.

**Figure 5:**
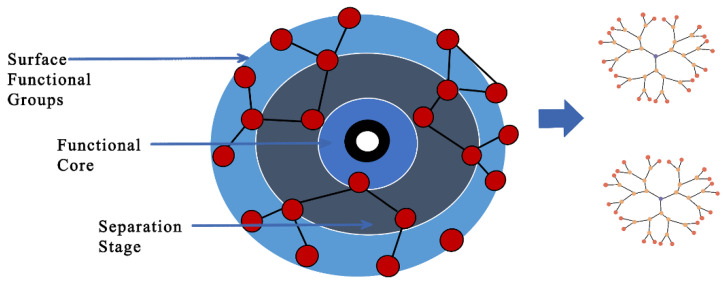
Illustration of dendritic molecular structures with a central core, repeating branches, and terminal reactive functional groups. They can be classified as polymers and hyperbranched polymers with convergent and divergent architectures, depending on their molecular nature weight. Their nanostructure provides dendrimer–drug conjugation via different interactions such as electrostatic and hydrophobic/hydrogen bonds or the capacity for drug encapsulation within the central cavity and/or between the dendrons (branches). Adapted and modified from [87] under the Creative Commons license.

**Figure 7:**
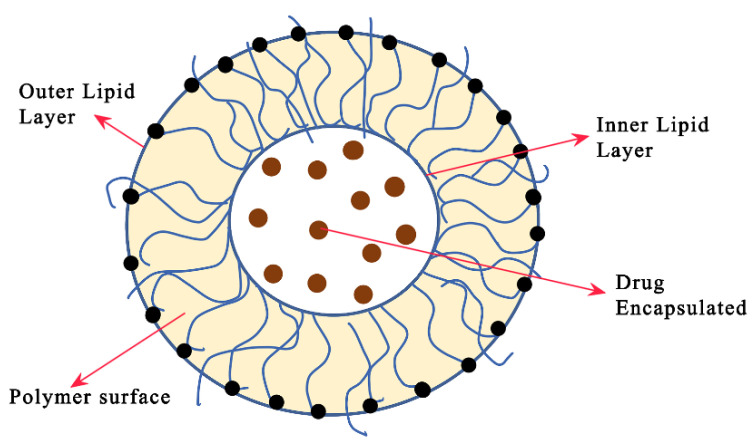
Illustration of a bilayer lipid-based nanocarrier with encapsulated drugs in the core; the self-assembled supramolecular architecture is shown. The solid lipid matrix encapsulates bioactive components, particularly lipophilic molecules, and releases them gradually over time. Lipid polymer nanoparticles typically have spherical particles and sizes in the range of 10 to 1000 nm. There are several forms of lipid-based nanocarriers (liposomes and niosomes) reported in reference [81] for drug delivery.
